# Genomic mapping reveals cisplatin disruption of protein phosphorylation signalling genome-wide

**DOI:** 10.1093/mtomcs/mfag016

**Published:** 2026-06-05

**Authors:** Luyu Qi, Qun Luo, Yinzhu Hou, Yan Xu, Wanchen Yu, Xingkai Liu, Bobo Xin, Yaolong Huang, Xiangjun Li, Yanyan Zhang, Shijun Wang, Peter J Sadler, Yao Zhao, Fuyi Wang

**Affiliations:** Beijing National Laboratory for Molecular Sciences, CAS Research/Education Center for Excellence in Molecular Sciences, National Centre for Mass Spectrometry in Beijing, CAS Key Laboratory of Analytical Chemistry for Living Biosystems, Institute of Chemistry, Chinese Academy of Sciences, Beijing 100190, China; University of Chinese Academy of Sciences, Beijing 100049, China; Beijing National Laboratory for Molecular Sciences, CAS Research/Education Center for Excellence in Molecular Sciences, National Centre for Mass Spectrometry in Beijing, CAS Key Laboratory of Analytical Chemistry for Living Biosystems, Institute of Chemistry, Chinese Academy of Sciences, Beijing 100190, China; University of Chinese Academy of Sciences, Beijing 100049, China; Beijing National Laboratory for Molecular Sciences, CAS Research/Education Center for Excellence in Molecular Sciences, National Centre for Mass Spectrometry in Beijing, CAS Key Laboratory of Analytical Chemistry for Living Biosystems, Institute of Chemistry, Chinese Academy of Sciences, Beijing 100190, China; University of Chinese Academy of Sciences, Beijing 100049, China; University of Chinese Academy of Sciences, Beijing 100049, China; Basic Medical College, Shandong University of Chinese Traditional Medicine, Jinan 250355, China; Beijing National Laboratory for Molecular Sciences, CAS Research/Education Center for Excellence in Molecular Sciences, National Centre for Mass Spectrometry in Beijing, CAS Key Laboratory of Analytical Chemistry for Living Biosystems, Institute of Chemistry, Chinese Academy of Sciences, Beijing 100190, China; NGS Department, Sangon Biotech (Shanghai), Shanghai 201611, China; NGS Department, Sangon Biotech (Shanghai), Shanghai 201611, China; University of Chinese Academy of Sciences, Beijing 100049, China; Beijing National Laboratory for Molecular Sciences, CAS Research/Education Center for Excellence in Molecular Sciences, National Centre for Mass Spectrometry in Beijing, CAS Key Laboratory of Analytical Chemistry for Living Biosystems, Institute of Chemistry, Chinese Academy of Sciences, Beijing 100190, China; Basic Medical College, Shandong University of Chinese Traditional Medicine, Jinan 250355, China; Department of Chemistry, University of Warwick, Coventry CV4 7AL, United Kingdom; Beijing National Laboratory for Molecular Sciences, CAS Research/Education Center for Excellence in Molecular Sciences, National Centre for Mass Spectrometry in Beijing, CAS Key Laboratory of Analytical Chemistry for Living Biosystems, Institute of Chemistry, Chinese Academy of Sciences, Beijing 100190, China; University of Chinese Academy of Sciences, Beijing 100049, China; Beijing National Laboratory for Molecular Science, Huairou Research Center, Institute of Chemistry, Chinese Academy of Sciences, Beijing 100190, China; Beijing National Laboratory for Molecular Sciences, CAS Research/Education Center for Excellence in Molecular Sciences, National Centre for Mass Spectrometry in Beijing, CAS Key Laboratory of Analytical Chemistry for Living Biosystems, Institute of Chemistry, Chinese Academy of Sciences, Beijing 100190, China; University of Chinese Academy of Sciences, Beijing 100049, China; Basic Medical College, Shandong University of Chinese Traditional Medicine, Jinan 250355, China; National Centre for Mass Spectrometry, Beijing, Beijing 100190, China

## Abstract

Cisplatin is a DNA-targeting chemotherapeutic. Here we investigate how the cisplatin-damaged gene (CDG) loci are linked to specific protein-driven signalling pathways. A human high mobility group protein 1 box a-based affinity probe has been constructed and 1,2-cisplatin-crosslinked DNA has been isolated before high throughput gene sequencing. Cisplatin damage to specific genes has been mapped in human lung cancer cells, and a total of 16 216 CDGs mapped with fold-enrichment >1.5. Surprisingly, bioinformatics analysis demonstrates that cisplatin targets most of the human protein kinase (PK) and phosphatase genes and is involved in 300 important cell signalling pathways (−log *p* > 4). The most associated key signalling pathways are sperm motility and protein kinase A. Notably, cisplatin damaged 85% (440) of human PK genes and 81% (110) of human protein phosphatase genes. This implies that cisplatin may disrupt protein phosphorylation signalling genome-wide, evidenced by a significant decrease in expression of a series of key PK genes.

## Graphical Abstract

**Graphical Abstract:**
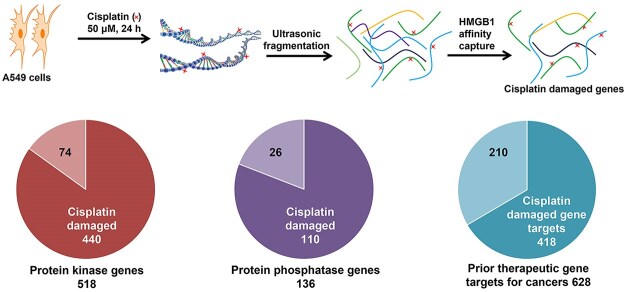

## Introduction

Cisplatin, *cis*-[PtCl_2_(NH_3_)_2_], is a first-line chemotherapeutic drug, in particular for systemic administration in combination therapy for solid tumours [1,2]. The major target is believed to be nuclear DNA (nDNA) [3,4], although the formation of nDNA adducts is not the sole determinant of cisplatin-induced cytotoxicity, [5] it has been linked to side effects and toxicity [6]. The formation of 1,2-intrastrand -GG-/-AG- (90%) adducts, 1,3-intrastrand -GNG- adducts, and interstrand crosslinks unwinds and bends DNA helices, inhibiting DNA replication and transcription, and in turn inducing cell apoptosis and death [3].

The human high mobility group box 1 (HMGB1) protein is well recognized for its specific binding to cisplatin-crosslinked double-stranded DNA [4,7–9]. HMGB1 functions as a protector of cisplatin-induced DNA damage from nucleotide excision repair [4], and may serve as a negative transcription cofactor to block the binding of transcription factors such as Smad3 to DNA, as revealed by single-cell imaging [10]. Shu et al. developed a “cisplatin-seq” method to map cisplatination sites with a single base resolution throughout the whole genome [11]. They revealed that cisplatin was more likely to attack mitochondrial DNA (mDNA) than nDNA, and that the cisplatin damage was enriched within promotors and regions harbouring transcription termination sites [11]. In addition to the cisplatin-seq approach described above, Hu et al. have developed a set of methods, termed “damage-seq” and “eXcision Repair-seq” (XR-seq), to map, at single-nucleotide resolution, the location and repair of cisplatin lesions in genomic DNA adducts [12], respectively. Their mapping data demonstrated that cisplatin damage was uniform and dominated by the underlying sequence, but the repair of cisplatin lesions was heterogeneous throughout the whole genome and affected by multiple factors such as transcription and chromatin states. However, similar to the cisplatin-seq approach, the damage-seq method was not able to assign cisplatin lesions to specific gene loci. To address this issue, the same group integrated their “damage-seq,” “XR-seq,” and RNA-seq approaches to study comprehensively the genome-wide profiles of cisplatin lesions in mouse organs [13]. This revealed that the expression of 12 genes was commonly downregulated due to cisplatin damage across mouse kidney, liver, lung, and spleen [13]. However, the combined use of transcriptomics strategies did not produce a full map of cisplatin lesions throughout the whole genome due to the tissue specificity of transcriptomics.

Recently, Yue et al. summarized state-of-the-art multiomics approaches for the exploration of cisplatin induced resistance, showing that mechanism studies from genome, transcriptome, proteome, metabolome, and epigenome provide novel information at different cellular function levels [14]. Sun et al. applied transcriptomics analysis for the cisplatin damage response genes and discovered that EGLN3 gene is key to induce chemotherapy resistance through TGF-β pathways [15]. Torres-Pineda et al. profiled gene expression for the cisplatin-treated rat brain and explored possible mechanisms of neurotoxicity [16]. However, this work was only hypothetical and lacked causal relationships. Very recently, protein kinase (PK) membrane-associated tyrosine/threonine 1, a member of the Wee kinase family, has been reported to be a key regulator of cisplatin sensitivity in osteosarcoma [17], suggesting that genes, especially kinase genes, may play important roles in the mechanism of action and resistance of this classic anticancer drug, cisplatin.

**Figure 1:**
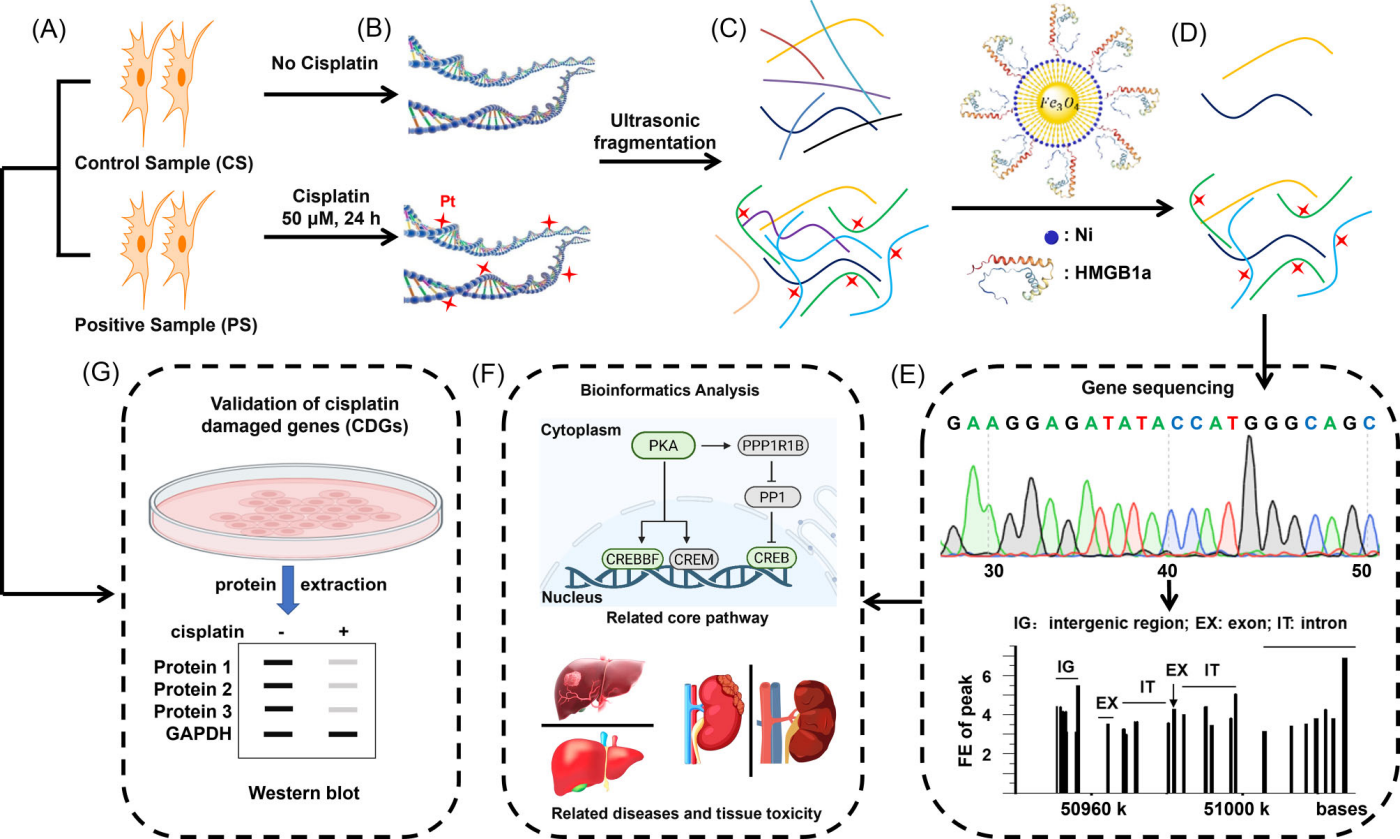
Diagrammatic illustration of the workflow for capture and mapping of gene loci attacked by cisplatin. The procedure includes (A) cell culture, (B) platination, (C) extraction, and fragmentation, and (D) capture of genomic DNA. (E) Sequencing and alignment of cisplatin-damaged DNA fragments. (F) Bioinformatics analysis (identification of CDGs). (G) Validation of CDGs by Western Blot assays.

The aim of the research reported here was to map the exact genomic distribution of cisplatin-damaged gene (CDG) loci by using a forward chemical genetics strategy [18,19]. Here, the HMGB1 box-a (HMGB1a)-based affinity microprobes and high-throughput sequencing have been combined to screen gene-lesion loci induced by cisplatin on a whole-genome scale (Fig. 1), and to correlate the gene lesions with the action of the drug. Full bioinformatics analysis of CDGs revealed that cisplatin disrupts the phosphorylation and dephosphorylation of proteins genome-wide. Protein phosphorylation and dephosphorylation are known to be critically important in cancer progression [20].

## Results

First, an HMGB1a-based affinity probe was constructed to enrich cisplatin-damaged DNA fragments generated by sonication of DNA from human A549 nonsmall cell lung cancer cells treated with cisplatin. Second, cisplatin-damaged DNA fragments were sequenced to map the damaged gene loci. Third, bioinformatics analysis was performed using the ingenuity pathway analysis (IPA) program to correlate CDGs with core signalling pathways (CSP), diseases and toxicity. In addition, the impact of cisplatin damage on the expression of protein-encoded genes was validated using Western Blotting.

### Enrichment of cisplatin-damaged DNA

HMGB1 protein has been shown to have high affinity to 1,2-cisplatin-crosslinked DNA [3,7,8,21], and HMGB1-based probes have been applied successfully to capture cisplatin-crosslinked DNA fragments [11]. In this work, we functionalized Ni-based magnetic microbeads with HMGB1a to assemble affinity microprobes for capturing and isolating 1,2-cisplatin-crosslinked DNA from genomic DNA fragments derived from A549 cancer cells treated with 50 μM cisplatin for 24 h for high-throughput Next Generation Sequencing (Fig. 1 and Experimental Section). The incubation conditions were optimized to ensure significant platination of nuclear DNA whilst retaining an adequate number of living cells. This optimized condition is consistent with that suggested by our previous work [10]. Details of the expression and purification of HMGB1a, construction of affinity microprobes, cell harvesting, cisplatin-damaged DNA extraction, fragmentation, and sequencing are described in the Experimental Section in the Supplemental Materials and summarized in Fig. 1.

### Sequencing and validation of genes

**Figure 2:**
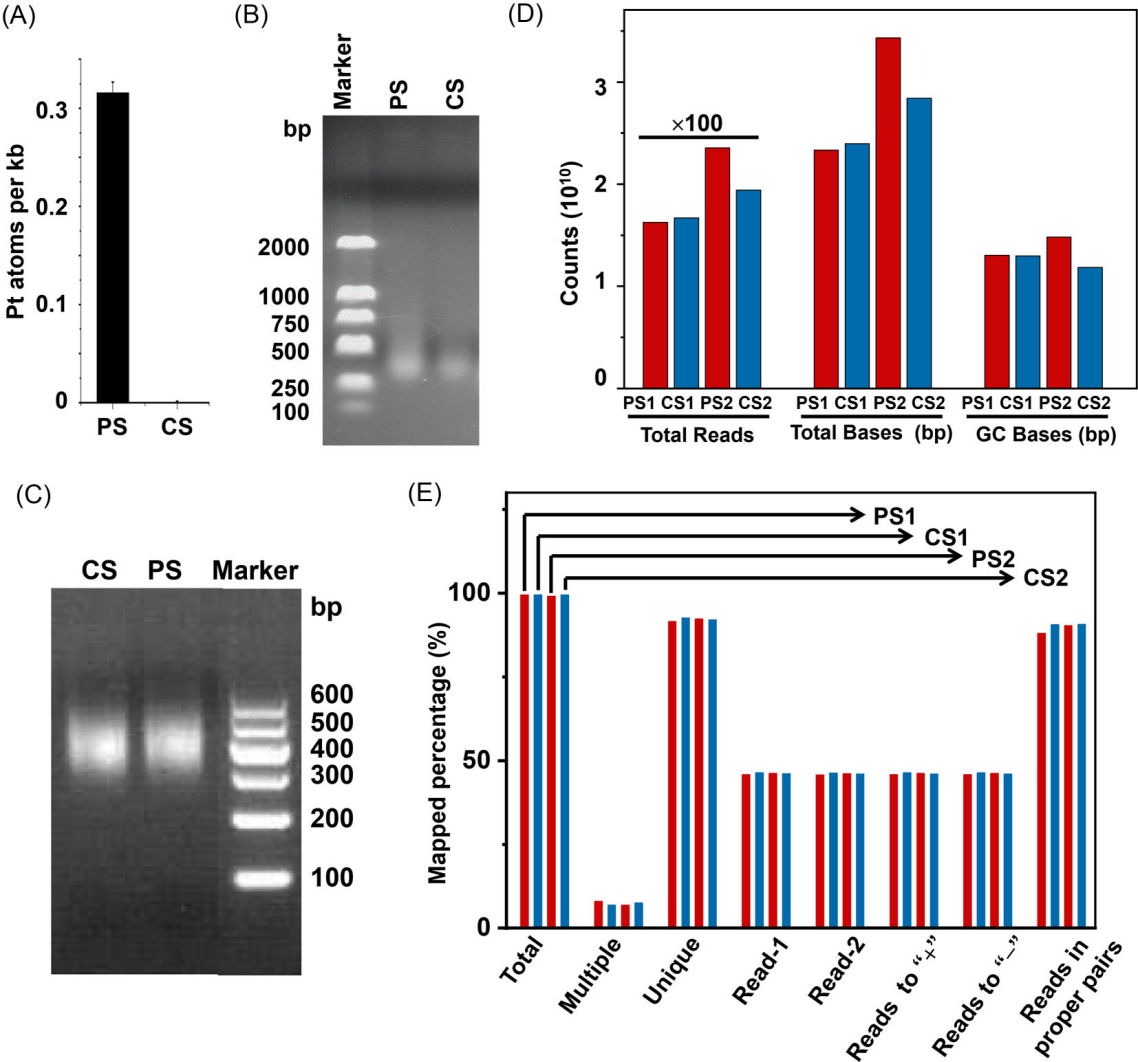
Mapping of CDGs. (A) The platination level of DNA fragments extracted from PS and CS, determined by ICP–MS. (B) The size distribution of DNA fragments from PS and CS groups. (C) The size distribution of sequences in DNA libraries constructed from captured DNA fragments. (D) Counts of total read, total bases, and GC bases mapped to PS and CS groups. (E) Percentage of mapped genes for PS and CS groups. Total: total mapped genes; multiple: multiple mapped genes; unique: unique mapped genes; read-1 & 2: first and second read; reads to “+” & “−”: read from 5′- to 3′-end (+) and 3′- to 5′-end (−); reads in proper pairs: reads with proper base-pair matching.

Since HMGB1 binds to cisplatin-crosslinked DNA with high affinity [7,21], but can also bind to bent native DNA [3], we extracted and fragmented genomic DNA from A549 cells with (positive samples, PS) and without (control samples, CS) exposure to cisplatin for DNA sequencing. These DNA fragments were then subjected to high-throughput sequencing after determination of the platination level (Fig. 2A) and quality check (Fig. 2B and C).

We minimized the risk of false negative results from gene-mapping, as described in the Experimental Section in the Supplemental Materials (Sequencing and gene-mapping). The total read counts and total base counts of the PS did not show a pronounced difference from those of the CS (Fig. 2D). However, because cisplatin preferentially crosslinks -GG- sites on DNA, the GC base-pair counts of the PS accounted for a slightly higher ratio to the total base counts than those of the CS (Fig. 2D; Supplementary Table S1). Consequently, the mapped percentage of total reads in properly-matched base pairs of PS were slightly lower than those of CS (Fig. 2E; Supplementary Table S1). Nevertheless, there are no significant differences in the total mapping percentage (≈99%) and unique mapping percentage (>91%) of genomic DNA fragments from both PS and CS. These data show that the quality of the sequencing is reliable to characterize CDGs throughout the whole genome.

### Mapping of CDGs

**Figure 3:**
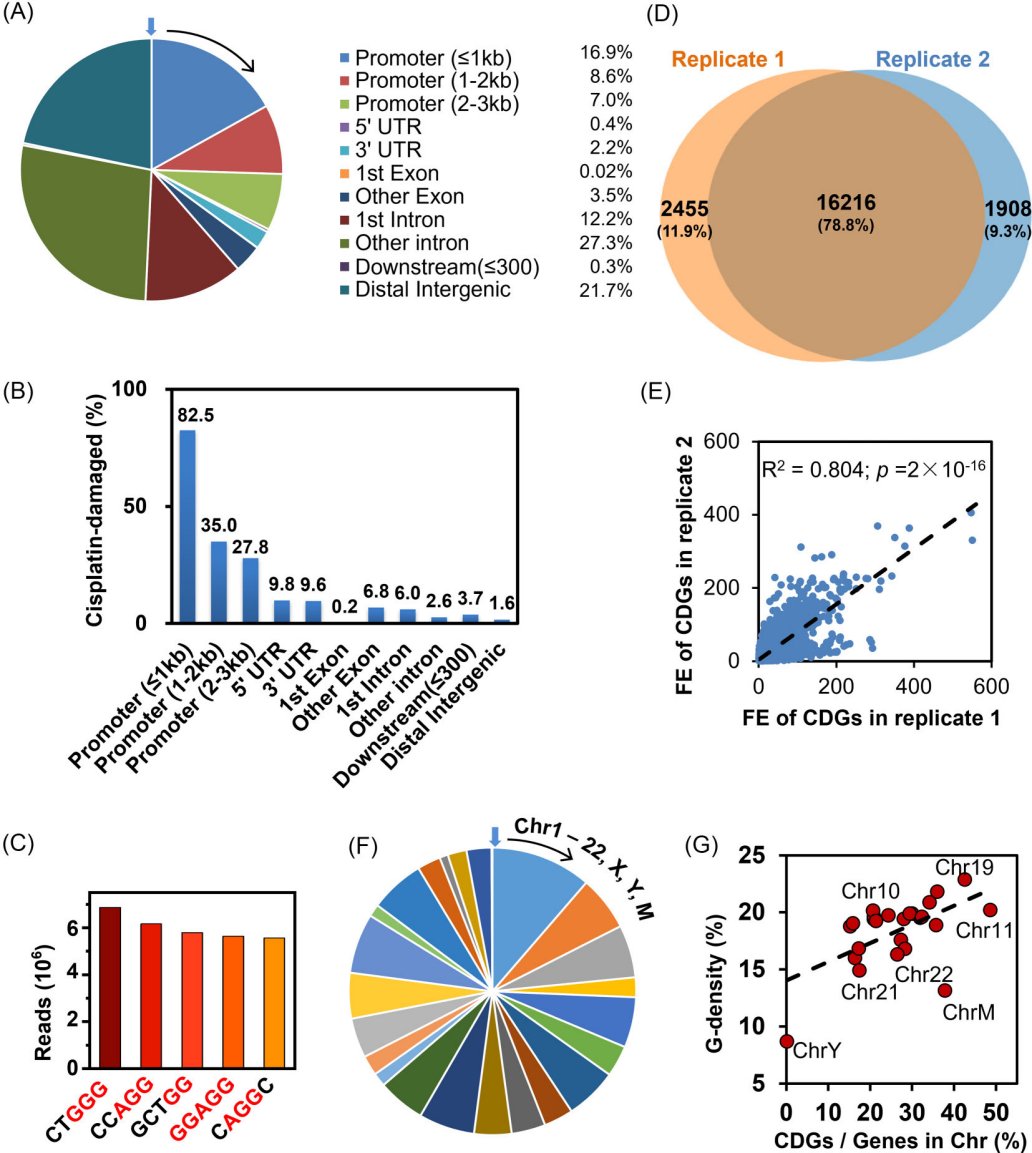
(A) Distribution of cisplatin-damaged DNA fragments in genomic regions. (B) Percentage of the lengths of the cisplatin-damaged DNA fragments in specific genomic regions. (C) The five pentanucleotide motifs that were the most frequently detected in CDGs. (D) Venn Diagram of total CDGs with FE_G_ > 1.5 in two independent biological replicates. (E) Correlation analysis of the normalized FE_G_ of CDGs in two replicates. (F) Distribution of CDGs over 23 chromosomes and mitochondria (Extended Table 3). (G) Correlation of the relative G-density in the chromosome (*y*-axis) with the proportion of CDGs detected in a chromosome to total number of genes in the human genome (*x*-axis).

To identify CDGs, we compared the total reads of all peaks mapped to a gene in the PS with those of all peaks mapped to the same gene in the corresponding CS. If the ratio [number of peaks]_PS_/[number of peaks]_CS_, the fold-enrichment of a peak (FE_P_) mapped to a gene, was ≥ 1.5, the peak was counted to the gene [22–25]. The fold-enrichment of a gene (FE_G_) is the sum of the FE_P_ of all peaks mapped to the gene (details in Materials and methods in the Supplementary Materials). Notably, to eliminate the bias arising from the different total reads of all peaks mapped in all samples, the normalized value of the read of a peak to the total read of all peaks for the sample was used (Supplementary Table S1). Following this method, we mapped 246 424 and 362 594 peaks with FE_P_ ≥ 1.5 from replicates 1 and 2, respectively. As shown in Fig. 3A, the peaks mapped to promoter regions, located 3k base pairs upstream of a gene, account for the highest percentage (32.48%) of all peaks mapped genome-wide. Considering the different lengths of each region in the genome, the coverage of peaks mapped to the promotor regions of all CDGs accounts for 82.5% of the total length of the promotor region 1 (≤1 kb), 35.0% of the promotor region 2 (1 kb), and 27.8% of the promotor region 3 (2–3 kb) (Fig. 3B). These coverages are much higher than for peaks mapped to other regions, e.g. exon and intron regions. These results show that cisplatin damage is enriched in the promotor regions, in particular, in regions close to encoding regions, in line with previous reports [11]. Since HMGB1a binds to double-stranded DNA over 5 base-pairs [3], the occurrence frequency of pentanucleotide motifs for all peaks containing platination sites was calculated, showing that the top five most-frequently platinated pentanucleotide motifs are CTGGG, CCAGG, GCTGG, GGAGG, and CAGGC (Fig. 3C). This is consistent with the well-established finding that cisplatin prefers to bind to GG sites on DNA [3,4].

When the FE of a CDG was calculated, the peaks mapped to the distal intergenic region of a gene were discarded. Following this procedure, a total of 16 216 CDGs with FE_G_ ≥ 1.5 [22,25] were mapped which were common in the two replicates (Fig. 3D; Supplementary Table S2). Moreover, the FE_G_ of CDGs mapped in the two replicates were linearly correlated, with R = 0.804, as shown in Fig. 3E, evidence of good reproducibility of the sequencing. The four CDGs with the highest FE_G_ mapped in this way are shown in Supplementary Fig. S1.

The 16 216 CDGs are unevenly distributed over all 23 pairs of chromosomes (Fig. 3F and Supplementary Table S3), showing a moderate dependence on the average G-density of the chromosomes, i.e. the higher the average G-density of a chromosome, the more the genes are attacked by cisplatin (Fig. 3G and Supplementary Table S3), again consistent with the known preference of cisplatin for crosslinking -GG- sites.

### Bioinformatics analysis

**Figure 4:**
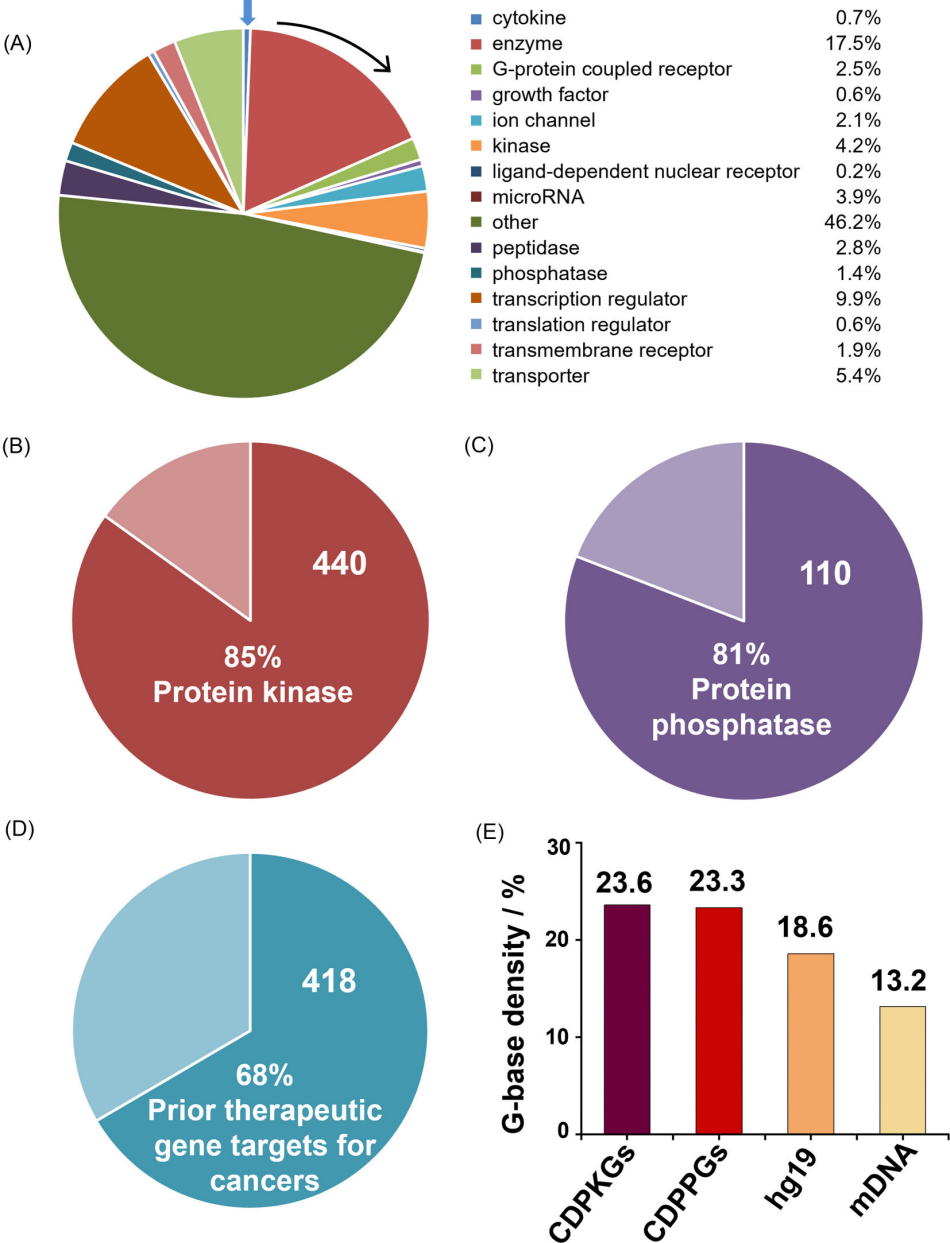
(A) Biological function classification of 14 971 protein-encoding CDGs with FE_G_ > 1.5. (B) Ratio of cisplatin-damaged protein kinase genes (CDPKGs) in all the 518 reported putative PK genes in the human genome [26]. (C) Ratio of cisplatin-damaged protein phosphatase genes (CDPPGs) in all the 136 reported protein phosphatases in the human genome [27]. (D) Ratio of CDGs in the 628 therapeutic gene targets for cancers identified by the CRISPR/Cas9-based screening [28]. (E) Average percentage of G bases in CDPKGs and CDPPGs, hg19, and mDNA.

The IPA program was employed to perform genome-wide annotation on the mapped CDGs. The 16 216 CDGs were input into the IPA data pool and matched to 14 971 protein-encoding genes in the human genome (hg19), including 2615 enzymes, 1488 transcription regulators, 810 transporters, 634 kinases, and 205 phosphatases (Fig. 4A, Supplementary Table S2). Eleven microRNA (miRNA) encoding genes were also identified, through these genes have a relatively low FE_G_ as their sequences are much shorter than genomic DNA.

Notably, among the 634 cisplatin-damaged kinase genes, 440 are PK genes (Supplementary Table S4), accounting for 85% of 518 reported putative PK genes in the human genome (Fig. 4B) [26]. There are also 110 cisplatin-damaged protein phosphatase genes (CDPPGs) among the 205 cisplatin-damaged phosphatase genes (Supplementary Table S5), accounting for 81% of the 136 reported protein phosphatases in the human genome (Fig. 4C) [27]. Significantly, among the CDGs, 418 genes belong to the 628 prior therapeutic gene targets for cancers identified previously by CRISPR/Cas9-based screening (Fig. 4D), 54 (13%) of which are targets of clinically-used drugs or candidates in preclinical development [28] (Supplementary Table S6). Also, the average percentages of G-bases in cisplatin-damaged protein kinase genes (CDPKGs) and CDPPGs are significantly higher than that of all the genes in the human genome (hg19, Fig. 4E). Therefore, platination on the whole genome is preferential for genes with high G-density, consistent with previous reports [3,4,11]. For lung cancer cells, only 14 cisplatin-damaged mitochondrial genes were identified with low FE_G_ ranging from 1.5 to 2.4 (Supplementary Table S7). Considering the much lower G-density in mDNA (Fig. 4E), it is reasonable that mitochondrial DNA has a low platination level.

It is notable that among the 16 216 CDGs with FE_G_ > 1.5, there are 36 pseudogenes (Supplementary Table S2). In general, pseudogenes are thought to be nonfunctional “junk genes.” However, increasing evidence argues that pseudogenes can act as key regulators at DNA, RNA, or protein levels in various human diseases such as cancer [29,30].

### Highly associated CSP (core signalling pathways) of CDGs

**Figure 5:**
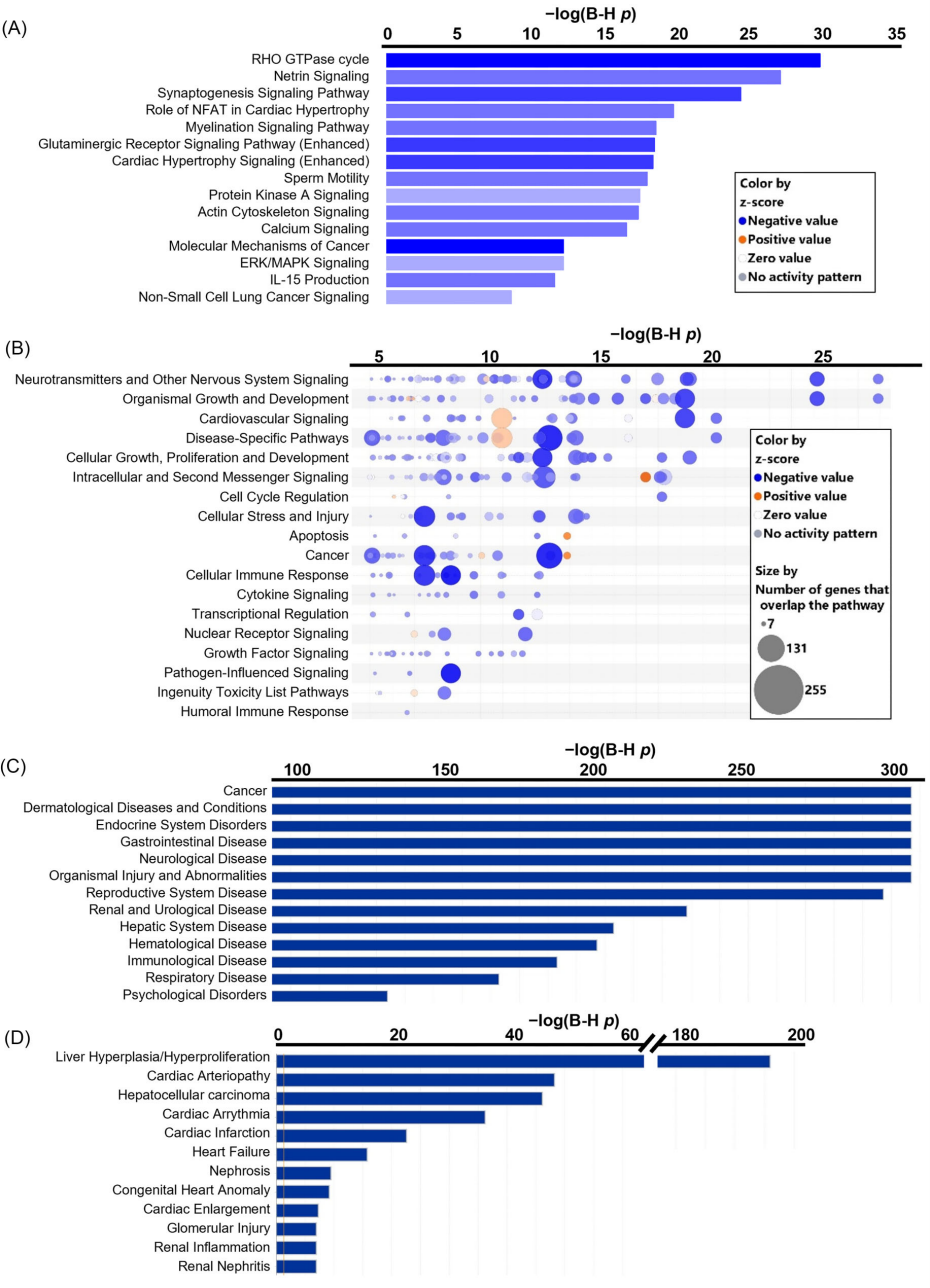
Bioinformatics analysis of CDGs. (A) The CSPs with which CDGs are associated. The details of these signalling pathways from top (RHO GTPase cycle) to bottom (non-small cell lung cancer) are provided in the Supplementary Figs. S2–S16, respectively. (B) The CDGs-associated CSPs can be further classified into 19 categories. (C) Diseases and functions with which CDGs are associated. (D) Toxicities with which CDGs are associated. B-H *p* means the *P*-values have been corrected by the Benjamini-Hochberg approach.

In order to analyze only the significant genes from the dataset and to input a reasonable number of molecules to IPA, the CDGs with FE_G_ > 20 (4774 genes) were selected and processed by IPA for the annotation and enrichment of CDGs-associated core signaling pathways. They are associated with 300 CSPs with -log(*p*) > 4, i.e. *p* < 0.0001 (Supplementary Table S8). The lower the *P*-value, the more associated the signalling pathways with the CDGs. The topmost associated pathways of these CDGs includes RHO GTPase cycle, netrin, synaptogenesis, myelination, glutaminergic receptor (enhanced), sperm motility (SP), actin cytoskeleton, ERK/MAPK, Rho family GTPases, and protein kinase A (PKA) signalling pathways (Fig. 5A and Supplementary Figs S2–S16).

All signalling pathways associated with the CDGs can be classified into 19 categories (Fig. 5B). Our analysis shows that cisplatin damage on genes affects a wide range of biological processes, such as organismal and cellular proliferation and development, cell cycle regulation, cellular stress and injury, apoptosis, etc. Other highly associated categories (with negative z-scores) such as intracellular and second messenger signalling, cancer, and cellular immune response, are also closely related to the inhibition of cancer cell growth. Also, some categories such as neurotransmitters and other nervous system signalling pathways, and cardiovascular signalling pathways, may be closely related to the known side effects of cisplatin, such as neurotoxicity and cardiotoxicity [6,31].

Furthermore, IPA revealed that among the top 300 CDG-associated CSPs (−log *p* > 4), CDPKGs on average account for 24% of the genes in each CSP (Supplementary Table S8). This proportion is much higher than that (4.8%) of CDPKGs to the 4774 CDGs with FE_G_ > 20 in the whole genome (Supplementary Table S4). This suggests that the damage on PK genes plays a significant role in the mechanism of action of cisplatin.

It is notable that CDGs are highly associated with PKA signalling pathway with −log *p* = 17.3 (Fig. 5A, Supplementary Fig. S12), and 152 CDGs including 28 (18.4%) CDPKGs, are involved in this signalling pathway (Supplementary Tables S8 and S9). Among the CDPKGs involved in PKA signalling pathway, a series of PKs showed high fold-enrichment (FE_G_ > 100), e.g. *PRKAG2*: 251, *PRKCE*: 161, *PRKCB*: 133, *PRKCZ*: 115, and *PRKAR1B*: 112, which indicates that PKs play crucial roles in the activity of cisplatin, meriting further investigation as a potential target for cancer treatment.

ERK/MAPK signalling is also an important pathway affected by CDGs (Supplementary Table S8, Supplementary Fig. S11). ERK/MAPK cascades regulate a wide variety of cellular processes, including proliferation, differentiation, apoptosis, and stress responses [32]. Moreover, among the CDPKGs involved in PKA signalling pathway, a series of PKs showed high fold-enrichment (FE_G_ > 100), e.g. PRKAG2: 251, PRKCE: 161, PPP2R2C: 136, PRKCB: 133, PLCG2: 124, PTK2: 114, and PRKAR1B: 112. These data indicate that ERK/MAPK signalling, and associated PKs play crucial roles in the activity of cisplatin.

Another CSP with which the CDGs are highly associated is the molecular mechanisms of cancer signalling pathway (−log *p* = 12.1) (Supplementary Table S8, Supplementary Fig. S14). 255 CDGs are involved in this pathway, and cover 66% of all genes in it. The very negative *z* score (−13.2) predicted by IPA demonstrated that these gene lesions induced by cisplatin downregulate the development of cancers. This is consistent with the clinical therapeutic effect of cisplatin.

**Figure 6:**
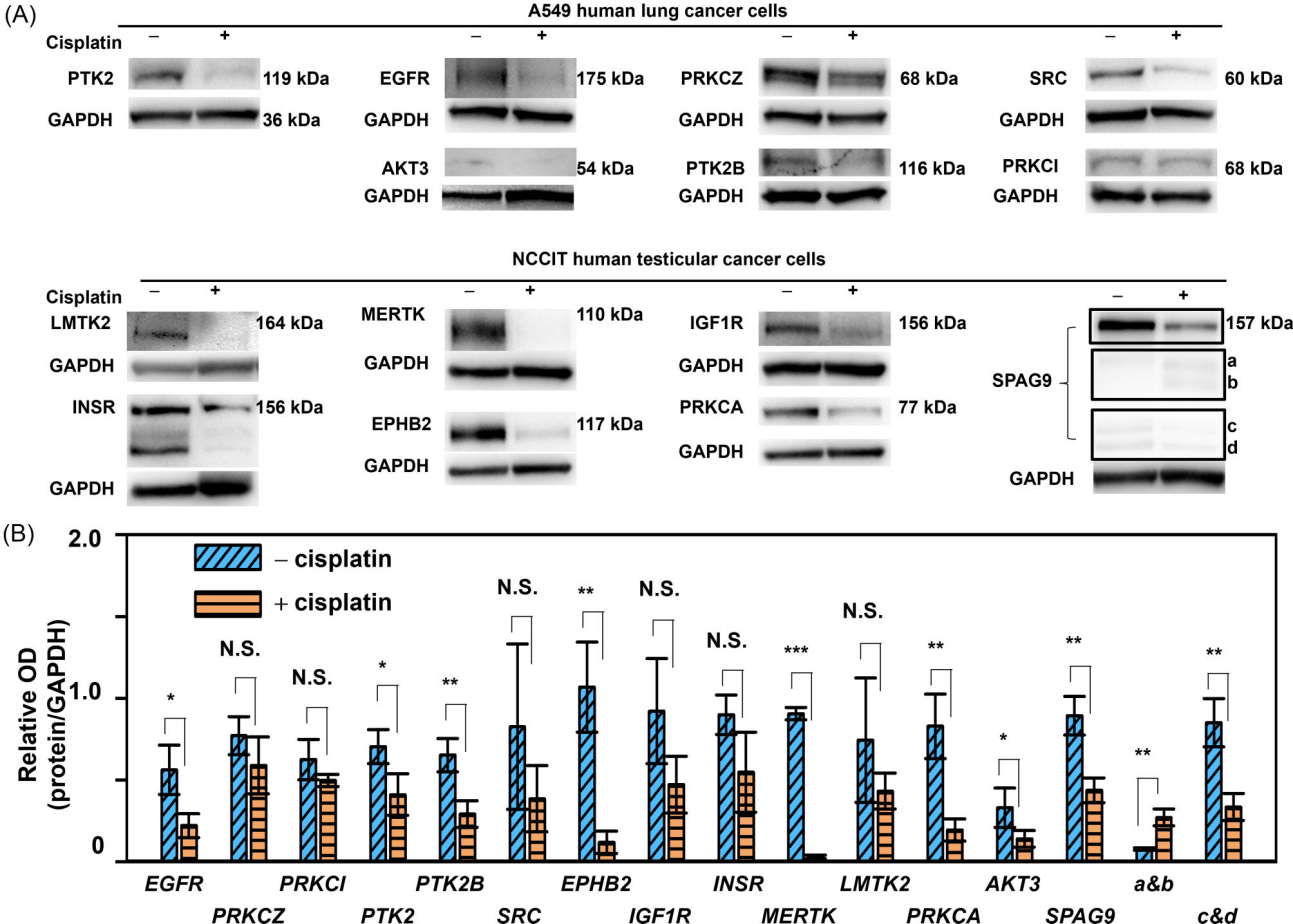
Western Blot assays and siRNA silencing. (A) Western Blot images of selected gene-encoded proteins expressed in A549 lung and NCCIT testicular cancer cells with (+) and without (−) cisplatin (12 μM) treatment. GAPDH (Glyceraldehyde-3-phosphate dehydrogenase) was the internal reference. The a–d subunits of JIP-4 (expressed by *SPAG9*) are shown in Supplementary Fig. S18A. (B) Optical density (OD) ratio of a gene-encoded protein to GAPDH. In B, two-tailed unpaired Student′s t-test was used to statistics process of the Western Blot assay (*n* = 3). N.S.: no significant; *P < .05, ** *P* < .01, *** *P* < .001. *p_EGFR_*= 0.0244, *p_PRKCZ_*= 0.2057, *p_PRKCI_*= 0.1615, *p_PTK2_*= 0.0366, *p_PTK2B_*= 0.0088, *p_SRC_*= 0.2343, *p_EPHB2_*= 0.0044, *p_IGF1R_*= 0.1000, *p_INSR_*= 0.0893, *p_MERTK_*= 2.71 × 10^−6^, *p_LMTK2_*= 0.2453, *p_PRKCA_*= 0.0062, *p_AKT3_*= 0.0126, *p_SPAG9_*_-full_ = 0.0049, *p_SPAG9_*_-a&b_ = 0.0030, *p_SPAG9_*_-c&d_ = 0.0061.

We found that CDGs are also highly associated with the non-small cell lung cancer (NSCLC) signalling pathway (−log *p* = 8.5) (Supplementary Table S8, Supplementary Fig. S16). Forty-seven genes, including 12 PKGs, of this CSP are damaged by cisplatin, covering 63% of all the genes in it. The *z*-score of this pathway is − 4.4, which therefore predicts to be significantly inhibited by cisplatin. Many patients with non-small-cell lung cancer have no response to the tyrosine kinase inhibitor gefitinib, which targets the epidermal growth factor receptor (EGFR) [33]. However, cisplatin can damage the *EGFR* gene in the NSCLC signalling pathway (Supplementary Table S2 and protein expression data shown in Fig. 6), which may play a role in the anticancer activity of cisplatin towards NSCLC. Given that AKT/PKB can promote survival of NSCLC [34], damage to the *AKT* gene by cisplatin may contribute to the killing of the NSCLC cancer cells.

We also found IL-15 production and SP signalling pathways, and a series of neural system and cardiac related signalling pathways. The details are shown in Supplementary Tables S8 and Supplementary Figs S2–S11, S13, and S15. The disruption to these signalling pathways by cisplatin may be related to the side effects of this drug.

### Association of CDGs with diseases and tissue toxicities

The association of CDGs with diseases and functions was analysed by IPA and the most associated disease of CDGs is cancer (Fig. 5C). The top five types of cancers with negative z-scores (predicting inhibition) to which CDGs are highly related are development of carcinoma (*z*-score −2.3), nonhematologic malignant neoplasm (*z*-score −3.8), abdominal carcinoma (*z*-score −3.6), abdominal adenocarcinoma (*z*-score −2.3), and digestive system cancer (*z*-score −3.9), (Supplementary Table S10, Supplementary Fig. S17). The negative *z*-scores for these cancers predict that they may be associated with CDGs.

It is notable that the sperm-associated antigen 9 (*SPAG9*) gene is involved in 147 of 361 cancers or functions with which the CDGs are highly associated (Supplementary Table S11). *SPAG9* abundantly expresses C-jun-amino-terminal kinase-interacting protein 4 (JIP-4) which consists of four subunits (Supplementary Fig. S18A) in testicular haploid germ cells and is essential for the development, migration, and invasion of cancer [35]. JIP-4 as a scaffolding protein knits mitogen-activated protein kinases (MAPKs) and their transcription factors to activate specific phosphorylation signalling pathways, such as MAPK and PI3K (phosphatidylinositol 3'-kinase) pathways [36]. These imply that cisplatin damage on *SPAG9* (FE_G_ = 62.0) may play a crucial role in the anticancer activity of cisplatin (confirmed by gene silencing and cell apoptosis experiments, as shown in Supplementary Fig. S21A–C).

Tissue toxicity is a major factor which limits the clinical application of cisplatin. Previous work on the toxic side effects of cisplatin have focused mainly on the organ and cellular uptake. Higher accumulation is usually closely related to higher toxic effects [6]. IPA annotation of the associated toxicity of cisplatin damage to genomic DNA suggests that the CDGs are closely associated with liver, heart, and kidney (Fig. 5D). The most highly related tissue toxicities are liver hyperplasia or hyperproliferation, cardiac arteriopathy, hepatocellular carcinoma, cardiac arrhythmia, cardiac Infarction, congenital heart anomaly, nephrosis, heart failure, cardiac enlargement, glomerular injury, renal inflammation, and renal nephritis (Fig. 5D and Supplementary Table S12). Since liver hyperplasia or hyperproliferation are usually related to liver tumours, liver carcinoma, and liver cancer, and the z-scores are negative (−2.5, −2.9, and −3.4, respectively), this result suggests that CDGs may be related to therapeutic effects. Previous clinical reports have shown that cisplatin accumulates mostly in liver and kidney rather than in heart [6,37,38], so the most relevant toxicity to all CDGs is likely to be nephrotoxicity. Although, as yet, there is still no causal relationship between the DCGs and the toxic side effects, and whole-organ toxicity in vivo is not solely due the molecular mechanisms of DNA damage, this work suggests for the first time that phosphorylation signaling pathways disturbed by CDGs may also be related to the molecular mechanisms for the toxic side effects of this drug. However, further investigation is needed to correlate the gene lesions induced by cisplatin with the phenotypes of clinical nephrotoxicity.

### Protein expression in cisplatin-treated cancer cells

To investigate the relationship between the CDGs and protein expression, classical Western Blot assays were performed. The expression level of 15 PK genes involved in sperm mobility signalling pathway, with which the CDGs are highly associated (Fig. 5A), was studied with or without cisplatin exposure (details in Supplementary Table S13). *SPAG9* (sperm associated antigen 9) was also chosen because it expresses JIP-4 protein which activate a series of phosphorylation signalling pathways [36]. Since these chosen genes are expressed at different levels in different types of cells and the SPAG9 gene is not expressed in the A549 cell line, we selected two human cancer cell lines, A549 (non-small cell lung cancer cell line) and NCCIT (testicular cancer cell line) to perform Western Blot assays. Testicular cancer is curable by cisplatin.

As shown in Fig. 6A, the expression levels of 14 selected genes decreased after cisplatin treatment. The changes in expression levels of 8 genes, including 7 PK genes and *SPAG9*, were significant (*P* < .05) or very significant (*P* < .01) (Fig. 6B). The data indicate that these genes were indeed damaged by cisplatin, substantiating the gene-mapping results described above. However, there is no direct correlation between the fold-change in expression of a gene and the FE_G_ of a specific CDG, or the base-coverage of cisplatin-damaged peaks over a damaged gene (Supplementary Fig. S19). This is probably attributable to the complexity of the gene expression machinery (Supplementary Fig. S18B).

The expression of the receptor tyrosine kinase *MERTK* (c-mer proto-oncogene tyrosine kinase) was the most strongly inhibited (fold change = 32) due to cisplatin damage (Fig. 6A and B). This receptor phosphorylates Akt1-Y26, mediating Akt activation and survival signalling, which in turn drives oncogenesis and therapeutic resistance [39]. Thus, cisplatin damage to *MERTK* may be implicated in the mechanism of action of cisplatin. Hence, *MERTK* is also a potential therapeutic target [39,40].

The expression of *MERTK*-related gene *AKT3* (RAC-gamma serine/threonine-PK) was inhibited 3.5-fold by cisplatin (Fig. 6A and B). *AKT3* encodes a member of the AKT serine/threonine PK family. As a middle-stream regulator of IGF1 (insulin-like growth factor 1) signalling, AKT3 plays a key role in signalling and regulating cell survival in insulin signalling, angiogenesis, and tumour formation. Cisplatin-induced damage to this gene could trigger a series of downstream changes, inducing apoptosis and death of cancer cells [41].

## Discussion and remarks

Cisplatin is a genetically toxic anticancer drug, but its gene targets or precise gene loci are largely unknown, limiting understanding of the cellular impact of DNA damage induced by the drug. In this work we have mapped genome-wide cisplatin-damage to gene loci based on A549 cells. As all cells have very similar genomes, our sequencing data for cisplatin-damaged nuclear DNA is of more general applicability and not limited to active genes in A549 cells. We further elucidated the associated CSP as well as diseases and toxicity by bioinformatics analysis using the IPA programme.

Two methods, termed “cisplatin-seq” [11] and “damage-seq,” [12] respectively, have been previously developed to map the cisplatin-damaged sites in genomic DNA. The “cisplatin-seq” developed by Shu et al. discovered that in HeLa cells, mitochondrial DNA (mDNA) is more susceptible to cisplatin attack than nDNA [11]. However, in our work, only 14 mitochondrial genes were detected to be damaged by cisplatin with low fold-enrichment ranging from 1.5 to 2.4 (Extended Data Table S7). Herein, moreover, untreated A549 cells were used as controls to eliminate possible false positive results because HMGB1 protein may bind strongly to intact mitochondrial DNA which is natively bent [42,43]. Considering the much lower G-density in mDNA (Fig. 2L), it is reasonable to assume that mitochondrial DNA has a low platination level. In this work, we used a similar affinity probe (HMGB1a) to capture cisplatin-crosslinked DNA, thus the sensitivity of our method is as good as that of their work.

The “damage-seq” method developed by Hu et al. [12] demonstrated that cisplatin damage was uniform and dominated by the underlying sequence, but the repair of cisplatin lesions was heterogeneous throughout the whole genome. Transcriptomics has been used in combination with the “damage-seq” method to map specific CDG loci across mouse organs [13]. Due to the tissue-specificity of transcriptomics, only the genes transcribed in specific tissues could be mapped for cisplatin lesions. Their platinated DNA was purified by immunoprecipitation with an anticisplatin antibody, which provided very good specificity.

Both these methods achieved single-base (nucleotide) resolution mapping of cisplatination sites on specific genomic regions such as promotors, transcription termination sequences and intergenic regions. However, the precise genes damaged by cisplatin throughout the whole genome and the biological consequences of the gene damage were not determined. This determination was a major aim of the present work. There are several chemical genetic strategies, in particular chemical proteomic approaches, that can uncover gene targets for cisplatin. These have produced significant advances in elucidating mechanisms of drug action. However, similar to the transcriptomics approach, they do not fully reveal CDG loci throughout the whole genome because they were focussed on discovering gene targets of cisplatin *via* measuring the difference between gene expression in specific types of cancer cells or tumour tissues upon cisplatin exposure. For example, Jimenez and co-workers found that UGGT1, COL6A1, and MAP4 can be used as potential biomarkers for determining cisplatin response, by proteomic analysis of non-small cell lung cancer cells [44]. Also Wilmes et al. have used integrated omics techniques to study differentially-expressed proteins and related pathways for cisplatin nephrotoxicity [45].

In this work, by integration of gene sequencing with bioinformatics analysis, our data provide three innovative findings. First, cisplatin attacks 440 PK genes with FE_G_ > 1.5 (Supplementary Table S4), covering 85% of consensual PK genes in the human genome [26]. Second, cisplatin also induced damage on 110 protein phosphatase genes, accounting for 81% of the 136 reported protein phosphatases in the human genome (Supplementary Table S5). Last but not least, amongst the 14 971 identified cisplatin-damaged protein-encoding genes, 418 genes are in the 628 prior gene targets for cancer treatment reported recently [28] (Supplementary Table S6).

CDGs reduced the expression of a series of PK genes. Bioinformatics analysis revealed that they are associated in a series of important signalling pathways such as PKA (Fig. 5A, Supplementary Table S8), as verified by Western Blot assays (Fig. 6). PKs, accompanied by phosphatases, regulate protein phosphorylation, the most common and important protein post-translational modification, involved in many natural biochemical pathways and cellular processes [46]. Since abnormal protein phosphorylation is closely related to cancer development, PKs are major drug targets for cancer therapy [47]. A number of small molecule PK inhibitors, e.g. gefitinib, imatinib, and dasatinib [48], have been developed and approved for clinical treatment of various cancers. Given our findings that PK genes are highly susceptible to cisplatin attack, and that the cisplatin-damaged PK genes are highly associated with 320 CSP, our work strongly suggests that cisplatin attack on kinase genes may play an important role in its mechanism of action.

The cisplatin-damaged protein phosphatase genes include a series of protein tyrosine phosphatase receptors (PTPRs), such as *PTPRD/F/M/E/J/U/S/T*, with FE_G_ of > 50. This may explain why the intracellular response to cisplatin activates rather than inhibits Ataxia Telangiectasia mutated (ATM)/ATM and Rad3-related (ATR)/DNA- PK dependent pathways [49,50] while it induces damage on PK genes as described above. Taken together, cisplatin damage on PK and phosphatase genes is genome-wide, implying that cisplatin exerts its pharmacological functions genome-wide by disturbing the signalling of protein phosphorylation in cancer cells.

Our data suggest that mitochondrial genes might not be the major targets for cisplatin, possibly because mitochondrial genes are usually short and have a low density of guanine, the major binding site of cisplatin on DNA. This result is consistent with the widely accepted knowledge that nuclear DNA (nDNA) is the major target of cisplatin [3,4]. Some reports have suggested that mitochondrial DNA (mDNA) is more susceptible to cisplatin attack than nDNA [5,11]. In contrast, those studies used different cell types (HeLa ovarian cancer cells [11] and immortalized lymphocytes [12]), compared to A549 lung cancer cells used here, and different doses of cisplatin and times of treatment.

As shown in Supplementary Fig. S20A, *SPAG9* (JIP-4) activates JUN/MAP kinase signalling [36,51], and is associated with various types of cancers, e.g. anaplastic thyroid carcinoma [52], hairy cell leukaemia [53], and intestinal gastric adenocarcinoma [54]. It also plays a role in several important biological processes, such as fertility [55], differentiation of skeletal muscle cells [56], and differentiation of neurons [57]. Moreover, the *SPAG9* gene is regulated by ligand-dependent nuclear receptor ESR1 [58], transmembrane receptor CAV1 [59], transcription factor ZNE217 [60] and transporter BSG [61], and regulates mainly ERK, JNK, and p38 MAPK signalling [62] (Supplementary Fig. S20B). JIP-4 functions as a scaffold protein that structurally organizes MAPKs and mediates c-Jun-terminal kinase signalling. It has been demonstrated that *SPAG9* is associated with tumour growth, migration, and invasion in renal cell carcinoma [63]. Sinha et al. discovered that down regulation of *SPAG9* by siRNA silencing reduces growth and invasion potential of triple-negative breast cancer cells [64]. Because *SPAG9* specifically expresses the upstream mediator JIP-4 of p38 MAPK signalling in high abundance in testis [65,66], the cisplatin damage on *SPAG9* may account for its high cure rate for testicular cancer, which deserves further investigation.

In this work, gene damage mapping is based on studies of A549 human lung cancer cells. However, because the mapping was performed by capturing the cisplatin-damaged nuclear DNA, the cisplatin-damage on the genes which are transcriptionally silent in A549 cells could be mapped too. For example, PTPRN2 was identified as a CDG with a fold-enrichment of 32.09, but this gene is transcriptionally silent in A549 cells [67]. In other words, our mapping results are likely to apply genome-wide and not be limited to transcriptionally active genes in A549 cells.

Cisplatin is the most successful metallo-anticancer drug in the clinic. Over 50% of the chemotherapy treatments use cisplatin-related compounds. In contrast to many organic drugs, metallo-drugs are usually multitargeted, and it is essential to understand the nature of their target sites to elucidate their systems pharmacology [68]. It is evident from our work that cisplatin targets a very wide variety of kinase and phosphatase genes. However, at this stage, we cannot determine unambiguously if cisplatin damage to these genes is dependent on their expression status and/or chromatin accessibility. Nevertheless, this work provides novel insights into the mechanism of action of this drug and a basis for design of next generation of platinum anticancer drugs. New therapeutic strategies could involve drug delivery systems that target specific PK genes which play crucial roles in the development of particular cancers [69], and minimization of nonspecific attack on PK genes of healthy cells [2,20].

## Supplementary Material

mfag016_Supplemental_Files

## Data Availability

The raw sequencing data are deposited on Mendeley Data, and available on https://data.mendeley.com/datasets/k9sm56gj5y/1, https://data.mendeley.com/datasets/2tv7ntcgh6/1, https://data.mendeley.com/datasets/4zj3kz34c5/1, https://data.mendeley.com/datasets/dmyfkn7pnz/1, https://data.mendeley.com/datasets/dw3v4sm9nb/1, https://data.mendeley.com/datasets/xb6s7vzcwm/1, https://data.mendeley.com/datasets/75dyzmvbrz/1, https://data.mendeley.com/datasets/s7jbxvwf4z/1, and https://data.mendeley.com/datasets/fv6f9rpg5m/1. The flow cytometry data contained in this article are deposited on FlowRespository with a Repository ID of FR-FCM-Z45H on https://flowrepository.org/experiments/4273.
